# Myeloid-derived suppressor cells (MDSCs): what do we currently know about the effect they have against anti-PD-1/PD-L1 therapies?

**DOI:** 10.3332/ecancer.2023.1556

**Published:** 2023-06-05

**Authors:** Ronald Sergio Limón Tellez, Lucia Reynolds, Miguel A Piris

**Affiliations:** 1 Department of Oncology, University Social Security USS, Nº58 Colon Street, 10260 Santa Cruz, Bolivia; 2 Associate Medical Oncology and Research, OncoBolivia Specialized Center for Cancer Treatment, Nº236 Azucenas Street, Equipetrol, Santa Cruz, Bolivia; 3 Department of Oncology and Research, Clinic of The Americas, Nº5001 Sixth Ring Avenue and Beni Street, 10260 Santa Cruz, Bolivia; 4 Associate Medical Chief Pathology Service, Fundación Jiménez Diaz, Nº228040 Reyes Católicos Avenue, 2552 Madrid, España

**Keywords:** Myeloid-derived suppressor cells (MDSCs), tumour microenvironment, immunotherapy, PD-1 and PD-L1

## Abstract

Recent advances in cancer treatment such as PD-1/PD-L1 checkpoint inhibitors have prompted multiple research studies to determine all of the factors that influence response or failure to these new treatments. One of those identified factors is myeloid-derived suppressor cells (MDSCs). These cells were identified and described for the first time in 2007 in laboratory mice and cancer patients. Previous studies showed that a greater number of MDSCs was directly related to a greater tumour volume. There are two clearly identified subpopulations: Mononuclear-type myeloid-derived suppressor cells (M-MDSCs) and polymorphonuclear (PMN-MDSCs). These cell population subtypes play a very important role, depending on the type of cancer, since they have the particularity of expressing PD-L1, which interacts with PD-1, inhibiting the expansion of cytotoxic T lymphocytes, promoting resistance to these treatments.

## Introduction

Advances in molecular biology techniques and knowledge of the functioning of these genes in the field of oncology have allowed us to know the characteristics that promote the development, growth, migration and progression of a tumour in our body, however, this is not enough for a cancer to affect us, other factors are required, such as the tumour microenvironment (TME), which plays a fundamental role in the progression and resistance to cancer treatments such as immunotherapy. TME refers to the place where the tumour manages to establish itself for its development, acquires the capacity to produce metastasis, in addition to developing mechanisms for the evasion of the immune system. TME is a very complex structure whose main functions are to protect tumour cells from our immune system and also to assist in tumourigenesis. The main components of this TME are tumour-associated macrophages (TAMs), t-regulatory cells, cancer-associated fibroblasts and Myeloid-derived Suppressor Cells (MDSCs). This work is focused on MDSCs and their role against immunotherapy drugs. Immunotherapy and its advances began in 2011, where the benefit of using these drugs in modulating our immune system with therapies such as Anti-CDLA4, which was the first antibody directed against the CTLA-antigen, was really seen. Four found on the surface of cytotoxic T lymphocytes, this antigen acts as a negative regulator of the activation of this type of lymphocytes. Therefore, by inhibiting the signal of this antigen, we can stimulate the proliferation and activation of these lymphocytes, which facilitates the destruction of tumour cells. The advance and discovery of new mechanisms involved in the modulation and inhibition of our immune system gave rise to the development of new drugs such as anti-PD-1 and anti-PD-L1 directed against PD-1 and PD-L1, respectively, all these drugs are currently known as checkpoint inhibitors (ICI acronym in English) and the manipulation of these checkpoints have given favourable results in different oncological pathologies, however, and like other treatments, they do not always give favourable results and this is due to different factors disease-specific and host-specific, in this review we are going to focus on MDSCs and the role they play against these new anti-PD-1 and anti-PD-L1 drugs as a mechanism of resistance and possible dedicated treatments to modify the actions of these MDSCs. [1, 2]

## Objectives

The bibliographic review is to know all the information available on the function of MDSCs and how they influence the response to treatments with anti-PD-1/PD-L1 immunotherapy; in addition to potential treatments against MDSCs today.

## Methodology

For an adequate selection of literature, the Pubmed, MedLine and The Cochrane Library Plus databases were consulted, without date restrictions, in Spanish and English, gray literature. Four search criteria were applied: 1) Development of the PICO (population, intervention, control, and outcomes) research question; 2) Identification of relevant literature; 3) Selection of clinical practice guidelines (CPG) and health technology assessments and 4) synthesis of the results and conclusions of the review, as well as keywords such as TME , suppressor cells derived from myeloid, PD-1 and PD-L1. We found 149 articles related to the topic 39 abstract and 110 reviews after being reviewed and discarding duplicate works as well as those not related to the specific topic to be reviewed, 22 references were selected that met the selection criteria and. the purpose of the literature review.

## Results

**Figure 1.**
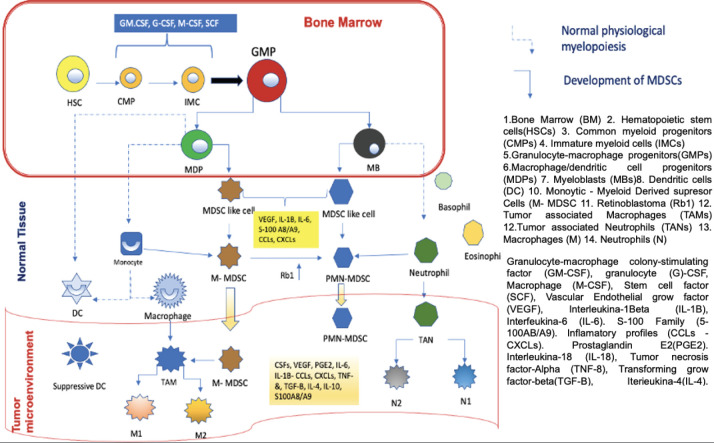
Development and progression of MDSCs in normal myelopoiesis. Based on Li *et al* [1] MDSCs as immunosuppressive regulators and therapeutic targets in cancer. Modified by Ronald Limón MD. MSc.

In order to understand the importance of MDSCs, we must first know where they come from, the functions they perform and, finally, how they interfere with the mechanism of action of the anti-PD-1/anti-PD-L1 control inhibitors. MDSCs come from haematopoietic stem cells (HSCs), for which it is necessary for inadequate or altered haematopoiesis to occur. This can occur in a situation where the host is exposed to a pathogen, leading to accelerated myelopoiesis in order to eliminate the offending agent as quickly as possible. In this phenomenon, myeloid cells are rapidly mobilised from the bone marrow (BM) to the place where the damage occurs, this process leads to a successive activation of certain molecular signaling components in response to the offending agent, within these components we have 1) Toll-like receptor (TLR) ligands 2) Damage associated molecular patterns 3) Pathogen associated molecular patterns. This successive activation of these different molecular compounds produces an increase in phagocytosis, respiratory burst, and upregulation of proinflammatory cytokines. This accelerated myelopoiesis process is temporary until the aggressor agent is eliminated, after which the myelopoiesis process returns to normal. However, in certain diseases such as cancer, autoimmune diseases, and nonspecific chronic inflammatory processes, this myelopoiesis is sustained in order to prevent persistent tissue damage. One of the main characteristics of cancer is to produce chronic inflammation in the host and in all the tissues where it is located, thus producing sustained myelopoiesis, in which immature myeloid cells (IMC) at a given time in the maturation process become aberrant, that is, they do not reach adequate maturation, obtaining different characteristics compared to normal myeloid cells. These immature cells have phenotypic and morphological characteristics of immaturity, weak phagocytic characteristics almost absent, in addition to altered anti-inflammatory and immunosuppressive functions, which is why we currently know them as MDSCs. These cells were studied and identified in mice in the laboratory and cancer patients for which common characteristics and certain differences were identified, this comparison helped to clearly identify this type of cells in humans. To classify the (MDSCs) we must take into account their morphology, density and phenotype thanks to this the MDSCs can be of two types 1) polymorphonuclear (PMN-MDSCs acronym in English) previously known as granulocytic and have a great resemblance to neutrophils; 2) monocytes (M-MDSCs acronym in English) very similar to monocytes, as we mentioned before it is necessary to describe the characteristics of these cells in the mouse and the differences of these cells to be identified in humans, for example, PMN-MDSCs cells Mouse cells label CD11b+Ly6G+ on their surface and M-MDSCs cells label CD11b+, Ly6C+, Ly6G−/low. In humans, PMN-MDSCs mark CD33dim, CD11b+, CD15+, CD14−, HLA-DR−/low and M-MDSCs are CD33+, CD11b+, CD14+, CD15−HLA-DR−/low, in some cases CD66b can replace CD15. Additionally, since humans have a complex karyotype, it was possible to identify a third subpopulation known as early-stage myeloid-derived suppressor cells. They are CD33+CD11b+ and their main characteristic is the absence of myeloid lineage markers. CD14, CD15 and CD66b. The absence or very low expression of HLA-DR on the surface of M-MDSCs in humans differentiates it from monocytes. It is very important that these cells are negative for lineage markers of T cells, B cells, and natural killer cells. PMN-MDSCs are very similar to neutrophils, so it is important to know how we can differentiate them. This was possible thanks to a complete analysis of the genome and flow cytometry examinations of these cells, thanks to which a specific marker of this could be identified. PMN-MDSCs cell type compared to neutrophils, this marker is lectin-like oxidised low-density lipoprotein receptor type 1 (LOX1) this labelling was seen in PMN-MDSCs from patients with head cancer and but in the same way, this test was performed on the neutrophils of the same patients with head and neck cancer, showing that the LOX 1 marker is specific for PMN-MDSCs cells and that it is never present in neutrophils, however, this analysis also revealed that at a certain point in the maturation of both cell groups they share the same phenotypic markers, but in the presence of a pathogen such as it is a tumour these myeloid cells acquire different characteristics and become immunosuppressive. [2, 3, 4]. Figure 1 uredescribes the development and maturation of HSCs and the differentiation process of MDSCs. In the BM, common myeloid progenitors derived from HSCs give rise to the expansion of granulocyte-macrophage progenitors (GMPs). GMPs differentiate into macrophages/dendritic cell progenitors (MDPs) and myeloblasts (MBs). All myelopoiesis is controlled by growth factors such as GM-CSF, G-CSF, M-CSF, and SCF. Under normal growth conditions (dotted line), MDPs expand and become macrophages and dendritic cells. (DC). MBs give rise to granulocytes, including basophils, eosinophils, and neutrophils. In pathological conditions such as cancer, most IMCs are pathologically activated and dedifferentiate into 2 cell types: M-MDSC and PMN-MDSC and in the presence of tumour-derived factors such as VEGF, IL- 6 and IL-1β, etc. In the early stages of the tumour, cells with biochemical characteristics similar to those of MDSCs do not have suppressive activity and are called MDSC-like cells. MDSCs can equally or partially arise from reprogramming existing differentiated monocytes and PMN-MDSCs . M-MDSCs can differentiate into PMN-MDSCs by transcriptional silencing of the retinoblastoma gene (Rb1). MDSCs cluster in peripheral tissues and the TME thanks to certain factors involved in chemotaxes, such as CCL2, CXCL and S100A8/A9. In TME, M-MDSCs have the ability to further differentiate into TAMs, TAMs can acquire M1 or M2 phenotypes, on the other hand, they can also differentiate into tumour-associated neutrophils and differentiate into tumour-inhibiting N1 and tumour-promoting subtypes N2.

### Role of MDSCs in tumour development and progression

One of the main characteristics of MDSCs is to alter immunity against a tumour; that is, to control the response of our immune system against the tumour by modifying the microenvironment where the tumour(s) are located, generating an immunosuppressive microenvironment, these changes that occur thanks to the activity of the MDSCs cause tumour progression and angiogenesis to also be promoted, but they also play a very important role in the development of resistance to antitumour therapies. In this case, we will refer to the mechanisms that occur for the development of resistance to treatments with anti-PD-1 and anti-PD-L1 and possible strategies to counteract this resistance.

### MDSCs as a mechanism of resistance against anti-PD-1/anti-PD-L1 therapies

**Figure 2.**
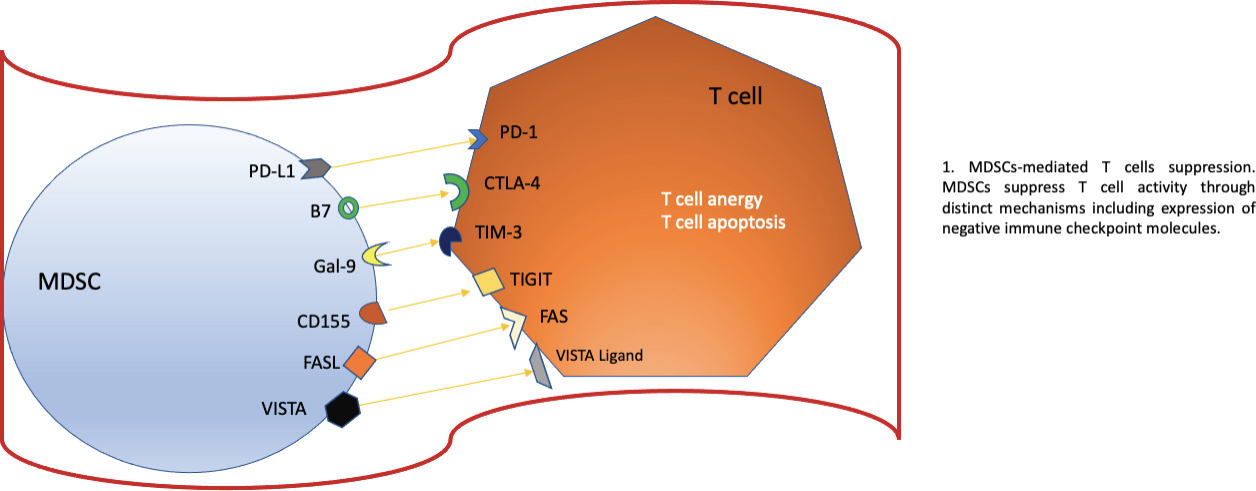
Negative regulators expressed by MDSCs. Based on Li *et al* [1] MDSCs as immunosuppressive regulators and therapeutic targets in cancer. Modified by Ronald Limón MD. MSc.

The objective of checkpoint inhibitor treatments (ICIs) is to modulate and modify the response that T lymphocytes have against the tumour, within these drugs we have the anti-PD-1 and anti-PD-L1 probably as the main ones and which have The natural evolution of many types of oncological diseases has changed, however, it is vital that for these targeted treatments to work, our lymphocytes are properly trained, that is, they have adequate functionality to attack the tumour. Hou *et al*. [8] described the mechanism of how MDSCs could induce resistance against anti-PD-1/anti-PD-L1 checkpoint inhibitor drugs. For these treatments to fail, an erratic functioning of our T lymphocytes must occur and how does this occur? Well, this occurs because the MDSCs induce a high concentration of Arginase (ARG1), decreasing L-Arginine uptake, which produces arrest of the cell cycle of T cells, inhibiting their expansion. Likewise, T cell dysfunction is produced due to the downward deregulation. of one of the chains of the T cell receptor (TCR for its acronym in English), this is due to the expression of the transporter Xc that produces a phenomenon where the MDSCs lose cysteine resulting in the blocking of the activation of the T cells due to to the limited availability of cysteine. However, there are other ways to alter this normal functioning or activation of T lymphocytes, one of which specifically affects TCRs, this is produced thanks to the hyperproduction of reactive oxygen species and peroxynitrite of MDSCs. MDSCs have the ability to upregulate PD-L1 on their surface, producing immunosuppression. PD-L1 is activated through the activation of IFN-alpha-STAT 1. IFN-alpha is highly expressed in cells of tumour tissues and, through phosphorylated STAT 1, binds to a sequence element of binding to the IRF pathway in vitro and to chromatin in vivo at the CD274 promoter to ultimately activate PD-L1 transcription in addition to IFN-alpha; IL-10, VEGF, and hypoxia are other critical modulators of PD-L1 expression in MDSCs. In the figure 2 we can see all the the negative regulators expressed by MDSCs after they have reached maturation. The expression of these receptors promotes a negative regulation and exerts a brake on our immune system, specifically affecting T cells.

### Active therapeutic targets against MDSCs

There are different strategies to control the action of MDSCs that are described below.

### Depletion of MDSCs

With the knowledge and functioning of these cells in the TME, it has been seen that there are certain chemotherapy drugs such as 5-fluorouracil capable of producing therapeutic effects in the tumour and in the MDSCs. this effect was observed in laboratory mice where 5FU induced tumour regression; In addition to a decrease in splenic MDSCs, it also induced a decrease in the neutrophil/lymphocyte ratio, which is probably related to a greater sensitivity to anti-PD-1/anti-PD-L1 immunotherapy treatments. On the other hand, in the TME, it is important to know that the vascular endothelial growth factor (VEGF) protein plays a very important role in the development of these immunosuppressive cells, since an expansion of these cells occurs through VEGF signaling. suppressor cells derived from myeloids in the TME this is due to the fact that there is greater recruitment of regulatory T cells, induces angiogenesis and promotes tumour progression; however, for a long time there have been different drugs directed against this VEGF protein such as bevacizumab or drugs targeting its VEGFR 1,2,3 receptor in its intracellular tyrosine kinase portion such as sunitinib used primarily in patients with kidney cancer for a long time as first-line therapy until 2019 where combinations of antiangiogenic drugs associated with anti-PD-1/anti-PD-L1 checkpoint inhibitors demonstrated significant improvements in their survival results. It is important to know the effects of these antiangiogenic drugs not only on the tumour but also on the TME, for example, sunitinib decreases MDSCs expansion due to the inhibition of the VEGF and c-KIT pathways. Additionally, it was also found that sunitinib inhibits STAT3 signaling, which is also related to the expansion of the MDSCs; consequently, in patients with kidney cancer treated with sunitinib, a decrease in MDSCs was seen as well as an improved function of T lymphocytes. Another drug that showed favourable results in preclinical studies was ibrutinib, currently used for the treatment of chronicle lymphocytic leukaemia.

### Induction of MDSCs maturation

A well-known drug used in acute promyelocytic leukaemia is trans-retinoic acid (ATRA). This drug promotes the maturation of a lineage of myeloid-derived cells. The mechanism of this drug led to investigate of what role it could have in other types of neoplasms such as kidney cancer, where CD33+ MDSCs were identified in blood samples, which were found in a higher concentration compared to healthy people. *In vitro* results from blood samples from kidney cancer patients showed that ATRA was able to induce the maturation and differentiation of these cells into antigen-presenting cell precursor cells, thus abrogating mediated immunosuppression. by MDSCs and optimising T cell function.

Other strategies against MDSCs, we know that these cells promote cell migration both inside and outside the TME, through the chemokine receptor CCR5, through the ligands CCL3, CCL4 and CCL5, plays a crucial role in the chemotaxis of the MDSCs, especially in the TME . Therefore, the blockade of CCR5 inhibits the recruitment and immunosuppressive activity of MDSC, this was evidenced in mice with melanoma where there was an improvement in their survival thanks to the blockade of CCR5. Similarly, CCR5 antagonists also inhibited the potential spread of triple-negative breast cancer and promoted decreased tumour growth; another important receptor is CSF-1R: which is a receptor tyrosine kinase that, when bound to its ligand CSF-1, promotes MDSC migration, differentiation and expansion of myeloid cells into MDSCs as well as TAMs. It has been seen that the CSF-1R receptor is deregulated, that is, elevated in several types of cancer, such as pancreatic and breast cancer. In preclinical studies, it has been seen that the inhibition of this pathway, either in its receptor or its ligand CSF-1R/CSF-1, increases and optimises the response of T cells. Weakening or attenuating the immunosuppressive properties of these cells also represents a great strategy to control the action of these cells here prostaglandin E2 (PGE2) also known as dinoprostone PGE2, positively regulates the elevation of ARG1 in MDSC, resulting in suppression of T cells; PGE2 was also found to increase Cyclooxygenase-2 (COX2) expression in monocytes, transforming them into M-MDSCs; Being a favourable mechanism for MDSCs, these cells have the ability to increase PGE2 production. The PGE2 suppressive function was documented in patients with melanoma, where it was discovered that the inhibition of COX2 downregulates the suppressive activity of M-MDSCs, as a consequence of this a greater decrease in tumour volume was observed, and finally, we will talk about the possibility of inhibiting the expression of PD-L1 and V-domain immunoglobulin suppressor of cell activation T (VISTA) in MDSCs, for this we need to know what mechanisms induce the expression of these molecules: a) PD-L1 is expressed in a large proportion in tumour -infiltrating MDSCs through IFNΓ, which is highly expressed in tumour tissue, with the pSTAT1-IRF1 pathway activating the PD-L1 promoter. b) Hypoxia and chronic inflammation induce the activation of NF-KB, which can also cause an increase in the expression of PD-1 and PD-L1 in MDSCs. c) The S-100 A9 gene is related to the increased expression of PD-1 and PD-L1 in MDSC, specifically in chronic lymphocytic leukemia (CLL). For example, when these patients with CLL received therapy with hypomethylating agents, an increase in expression was observed. of checkpoint proteins (PD-L1) in MDSC, concluding that it was due to a resistance attributed to a mechanism resistant to hypomethylation. d) Treg cells stimulate PD-L1 expression in MDSCs, we know that Treg cells stimulate PD-L1 expression in MDSCs from melanoma mice, on the other hand, the VISTA, also designated as PD-1H, is a well-established negative-type immune regulator now known to modulate immune evasion in cancer; Therefore, in addition to PD-L1, it is generally possible to find high levels of VISTA expression in MDSCs, monocytes and blasts of myeloid-type neoplasms, but this expression was not found in CD4+, CD8+ lymphocytes or Treg cells in the acute phase of this disease. leukaemic disease, this led to further research in different neoplasms such as lung cancer where it was seen that the high expression of VISTA/PD-1H is directly related to low tumour mutational load, observing the immunomodulatory role of VISTA/PD-1H these findings suggest that a combination of drugs against PD-1 and VISTA could induce more rapid and profound responses with immune checkpoint inhibitors (ICIs). In the table below we can see the different clinical studies directed against MDSCs in order to affect their development, proliferation and functioning through drugs such as chemotherapy, molecularly targeted therapy, immunotherapy. The multiple combinations with immunotherapy drugs such as anti-PD-1, anti-PD-L1 and anti-CTLA4 are also described. These combinations could not only promote a change in MDSCs in terms of their function or maturation but also promote greater sensitivity to immunotherapeutic treatment.

## Conclusion

MDSCs have widely demonstrated that they are capable of producing changes in the tumour microenvironment, promoting the development and progression of oncological disease; but probably the most important role today is resistance to checkpoint inhibitor treatments such as anti-PD-1 and anti-PD-L1. How is this produced? MDSCs are capable of inducing high concentrations of substances such as ARG1, which produces decreased L-Arginine uptake, inhibiting T-cell expansion as well as erratic T-cell function due to the limited availability of cysteine whose mechanism was described above however MDSCs have shown that they could be useful as biomarkers of resistance against anti-PD-1/anti-PD-L1 immunotherapy treatments, since a higher concentration of these cells at the start of treatment induces unfavourable results, despite all this in. We currently have drugs that have been shown to be capable of altering the functioning of these cells in order to promote better results in immunotherapy treatments, these drugs are some chemotherapy agents such as 5FU and/or derivatives, anti-angiogenic drugs such as sunitinib, both with different mechanisms produce a decrease in these myeloid suppressor cells, that is, they inhibit and decrease their expansion, resulting in a decrease in tumour volume and greater sensitivity to immunotherapeutic treatments; In addition, another drug active against these cells is ATRA, which induces the maturation of these cells and their differentiation, reducing their immunosuppressive properties and also promoting a better functioning of T cells. Finally, it is important to recognise the role that these cells play as mechanisms of resistance against anti-PD-1/anti-PD-L1 therapies and how possible combinations of treatments could help to obtain better results, such as anti-PD-1/anti-PD-L1 therapies associated with anti-angiogenic therapy used in different types of cancer according to certain molecular characteristics that promote their use, however, it is necessary to develop prospective studies that allow us to evaluate the role of these cells as biomarkers of response and selection of treatments in different types of neoplasms.

## Conflicts of interest and funding

The author is not aware of any affiliations, memberships, funding, or financial holdings that might be perceived as affecting the objectivity of this review.

## Figures and Tables

**Table 1.** Clinical trials evaluating MDSCs-targeting plus immunotherapy.

Target	Drug name	Combination therapy	Indication	
Promoting diffrentiation	STAT 3	AZD9150	MEDI4736	Malignant neoplasm of digestive, respiratory or intrathoracic organ
	STAT 3	AZD9150,AZD5069	MEDI4736 /Tremelimumab	Advanced solid tumours
	RAR/RXR	ATRA	Ipilimumab	Melanoma
	RAR/RXR	ATRA	Pembrolizumab	Melanoma
	RAR/RXR	ATRA,Cyclophospahmide	Vaccine	Lung cancer
	RAR/RXR	ATRA, Paclitaxel	Ad.p53-DC vaccine	SCLC
	TLR3	Poly ICLC	IMA 950	CNS tumour, Adult
	TLR7	Imiquimod	DC vaccine	Malignant glioma, Glioblastoma
	TLR9	CMP-001	Nivolumab	Melanoma, Lymph node cancer
	TLR9	CpG	Nivolumab	Pancreatic cancer
				
Inhibiting expansion and recruitment	GM-CSF	Sargramostin	Ipilimumab	Melanoma
	CSF-1R	Cabiralizumab	Nivolumab	Solid tumours
	VEGF	Bevacizumab, Entinostat	Atezolizumab	Metastatic cancer, Renal cancer
	VEGF	Bevacizumab, IPI-549	Atezolizumab	Breast cancer, renal cell carcinoma
	EGFR	Cetuximab	Ipilimumab	Head and neck cancer
	VEGFR	Cabozantinib, S-malate	Nivolumab + Ipilimumab	Thyroid cancer
	VEGFR	Regorafenib	Nivolumab	Hepatocelullar carcinoma
	IL-1B	Canakimumab	Spartalizumab	Renal cell carcinoma
	IL-8	BMS-986253	Nivolumab	Cancer
	CXCR1/2	Navarixin	Pembrolizumab	Solid tumours
	CXCR1/2	SX-682	Nivolumab	Colorectal cancer
	CXCR1/2	SX-682	Nivolumab	Pancreatic cancer
	CXCR1/2	SX-682	Pembrolizumab	Melanoma
	CCR2/CCR5	BMS-813160	Nivolumab	Pancreatic ductal adenocarcinoma
	CCR2/CCR5	BMS-813160	Nivolumab	Pancreatic ductal Adenocarcinoma
	CCR2/CCR5	BMS-813160	Nivolumab	Colorectal cancer, Pancreatic cancer
	CCR2/CCR5	BMS-813160, BMS-986253	Nivolumab	NSCLC, Hepatocellular carcinoma
	CCR5	Vicriviroc	Pembrolizumab	Colorectal neoplasms
	PI3K	IPI-549	Nivolumab	Cancer
				
Inhibiting Function	COX	Acetylsalicylic acid	Pembrolizumab	Head and neck cancer
	COX-2	Celecoxib	DC Vaccine	Ovarian cancer
	PDES	Tadalafil	Cancer Vaccine	Head and neck carcinoma
	PDES	Tadalafil	Telomerase Vaccine	Pancreatic adenocarcinoma
	NRF2	Omaveloxolone	Ipilimumab, Nivolumab	Melanoma
	HDAC	Entinostat	Nivolumab	Cholangiocarcinoma, Pancreatic cancer
	HDAC	Entinostat	Nivolumab	NSCLC
	HDAC	Entinostat	Nivolumab, Ipilimumab	Breast cancer
				
Depleting MDSCs		Gemcitabine	Nivolumab	NSCLC
		Gemcitabine	Modified vaccine	Ovarian cancer
		Gemcitabine	DC vaccine	Breast cancer
		Gemcitabine	DC vaccine	Sarcoma
		Capecitabine	Avelumab	Colorectal cancer
		Capecitabine - Cisplatin	Rituximab	Head and neck carcinoma
		Cyclophosphamide	iNKT cells, hrIL-2	Hepatocellular carcinoma
		Cyclophosphamide	Modified T cells	Leukaemia
		Cyclophosphamide	IMA970A plus CV8102	Hepatocellular carcinoma
		Cyclophosphamide	Tecemotide	Rectal cancer
		Cyclophosphamide, Fludarabine	Autologous transplant	Haematological malignancy
		Cyclophosphamide, Fludarabine	GD2-CAR-expressing Autologous T-lymphocytes	Neuroblastoma osteosarcoma
		Cyclophosphamide, Curcumin, Aspirin, Lansoprazole	Pembrolizumab	Cervical cancer, Endometrial cancer, Uterine cancer
		Docetaxel	DC Vaccine	Prostatic neoplasms
		Doxorubicin, Cyclophosphamide, Paclitaxel, Carboplatin, Decitabine	Pembrolizumab	Breast cancer
		Fluoruracil, Gemcitabine, Irinotecan, Oxaliplatin, Paclitaxel	Aldesleukin	Pancreatic cancer
		Vinorelbine	Atezolizumab	NSCLC
	MEK	Cobimetinib	Atezolizumab	Gallbladder carcinoma, Cholangiocarcinoma
	AKT	Ipatasertib	Atezolizumab	Solid tumoUr
	BTK	Ibrutinib	Nivolumab	Metastatic malignant solid neoplasm
	MET/VEGFR1/VEGFR/ROS1/RET/AXL/NTRK/KIT	Cabozantinib	Ipilimumab, Nivolumab	Large cell neuroendocrine carcinoma, Neuroendocrine carcinoma, small cell carcinoma
				
Inhibiting Metabolism	Liver-X-receptor	RGX-104	Nivolumab, Ipilimumab, Pembrolizumab	Malignant neoplasms
		Metformin	Pembrolizumab	Melanoma
	IDO	Epacadostat	Pembrolizumab	Melanoma
	IDO	BMS-986205	Nivolumab	Glioblastoma
	CD73	Oleclumab	Durvalumab	Pancreatic ductal Adenocarcinoma, NSCLC, Head and neck cancer
	CD73	Oleclumab	Durvalumab	NSCCLC, Renal cell carcinoma
	CD73	LY3475070	Pembrolizumab	Advanced cancer
	CD73	MEDI9447	MEDI4736	Triple-negative breast cancer
	CD73	MEDI9447	Durvalumab, Tremelimumab, MEDI 0562	Ovarian cancer
	CD73	MEDI9447	MEDI 4736	Solid tumours
	CD73	AK119	AK 104	Solid tumours
	CD73	Oleclumab	Durvalumab	Sarcoma
	CD73/A2AR	CPI-006 ciforadenant	Pembrolizumab	Cancer
				
Immunotherapy	PD-1	Nivolumab	Ipilimumab	Renal cell cancer
	CTLA-4	Ipilimumab	Nivolumab	Renal cell carcinoma
	CTLA-4	Ipilimumab	Nivolumab	Melanoma
	CTLA-4	Ipilimumab	Nivolumab	Acute myeloid leukaemia

Based on Li *et al* [1] MDSCs as immunosuppressive regulators and therapeutic targets in cancer and Clinical trials.gov. Modified by Ronald Limón MD, MSc
